# Early‐onset aging and mitochondrial defects associated with loss of histone acetyltransferase 1 (Hat1)

**DOI:** 10.1111/acel.12992

**Published:** 2019-07-10

**Authors:** Prabakaran Nagarajan, Paula A. Agudelo Garcia, Chitra C. Iyer, Liudmila V. Popova, William D. Arnold, Mark R. Parthun

**Affiliations:** ^1^ Department of Biological Chemistry and Pharmacology, The Ohio State University Columbus Ohio; ^2^ Department of Neurology The Ohio State University Columbus Ohio

**Keywords:** acetylation, aging, Hat1, histone, mitochondria, senescence

## Abstract

Histone acetyltransferase 1 (Hat1) is responsible for the acetylation of newly synthesized histone H4 on lysines 5 and 12 during the process of chromatin assembly. To understand the broader biological role of Hat1, we have generated a conditional mouse knockout model of this enzyme. We previously reported that Hat1 is required for viability and important for mammalian development and genome stability. In this study, we show that haploinsufficiency of Hat1 results in a significant decrease in lifespan. Defects observed in Hat1^+/−^ mice are consistent with an early‐onset aging phenotype. These include lordokyphosis (hunchback), muscle atrophy, minor growth retardation, reduced subcutaneous fat, cancer, and paralysis. In addition, the expression of Hat1 is linked to the normal aging process as Hat1 mRNA and protein becomes undetectable in many tissues in old mice. At the cellular level, fibroblasts from Hat1 haploinsufficient embryos undergo early senescence and accumulate high levels of p21. Hat1^+/−^ mouse embryonic fibroblasts (MEFs) display modest increases in endogenous DNA damage but have significantly higher levels of reactive oxygen species (ROS). Consistently, further studies show that Hat1^−/−^ MEFs exhibit mitochondrial defects suggesting a critical role for Hat1 in mitochondrial function. Taken together, these data show that loss of Hat1 induces multiple hallmarks of early‐onset aging.

## 1 INTRODUCTION

Aging is the major risk factor for many human diseases. Aging can be accelerated by defects in fundamental cellular processes such as mitochondrial function, DNA damage repair, and epigenetic inheritance (Benayoun, Pollina, & Brunet, 2015; Lopez‐Otin, Blasco, Partridge, Serrano, & Kroemer, 2013; Sen, Shah, Nativio, & Berger, 2016). Protein acetylation has emerged as a mechanism of regulation that may rival protein phosphorylation in importance (Choudhary et al., 2009; Choudhary, Weinert, Nishida, Verdin, & Mann, 2014; Drazic, Myklebust, Ree, & Arnesen, 2016; Norvell & McMahon, 2010; Xiong & Guan, 2012). The central role of protein acetylation in the regulation of aging was first uncovered through studies of the Sirtuin family of proteins (Haigis & Guarente, 2006). Sirtuins are NAD^+^‐dependent protein deacetylases. In organisms from yeast to mammals, alterations in Sirtuin function have been shown to modulate aging and lifespan (Kaeberlein, McVey, & Guarente, 1999; McDonnell, Peterson, Bomze, & Hirschey, 2015; Mostoslavsky et al., 2006; Rogina & Helfand, 2004; Tissenbaum & Guarente, 2001; Viswanathan & Guarente, 2011; Viswanathan, Kim, Berdichevsky, & Guarente, 2005).

Hat1 (or Kat1) was the first lysine acetyltransferase identified and serves as a paradigm for the study of type B histone acetyltransferases (Kleff, Andrulis, Anderson, & Sternglanz, 1995; Parthun, Widom, & Gottschling, 1996). Type B histone acetyltransferases are responsible for the acetylation of newly synthesized histone H4 prior to assembly into chromatin (Nagarajan et al., 2013). Loss of this modification on newly synthesized histone H4 translates into numerous alterations in nascent chromatin structure during DNA replication (Agudelo Garcia et al., 2017). In addition, high levels of spontaneous DNA damage, sensitivity to DNA damaging agents, and genome instability are highly conserved phenotypes in Hat1^−/−^ cells that are observed in fungi, chicken, and mammalian cells (Barman et al., 2006; Benson et al., 2007; Ge et al., 2013; Ge, Wang, & Parthun, 2011; Nagarajan et al., 2013; Qin & Parthun, 2002, 2006; Tscherner, Stappler, Hnisz, & Kuchler, 2012; Yang et al., 2013). Hat1 is also essential for viability in the mouse, as pups lacking Hat1 display neonatal lethality. In addition, neonates lacking Hat1 exhibit impaired lung development among other developmental abnormalities (Nagarajan et al., 2013).

To obtain a more complete view of the biological function of Hat1 in mammals, we have analyzed mice that are heterozygous for Hat1 (Hat1^+/−^). Strikingly, Hat1^+/−^ mice show a significant decrease in lifespan. Hat1^+/−^ mice display a number of pathologies that suggest this loss of viability is the result of early‐onset aging. Consistent with this, at the cellular level, Hat1^+/−^ cells display an increase in senescence markers. Further analyses indicate that, in addition to previously shown defects in chromatin structure and DNA repair, loss of Hat1 results in impaired mitochondrial function. Hence, Hat1^+/−^ mice are a novel model of premature aging that can provide insight into the role of protein acetylation in the regulation of multiple cellular processes.

## 2 RESULTS

### 2.1 Haploinsufficiency of Hat1 reduces lifespan

**Figure 1:**
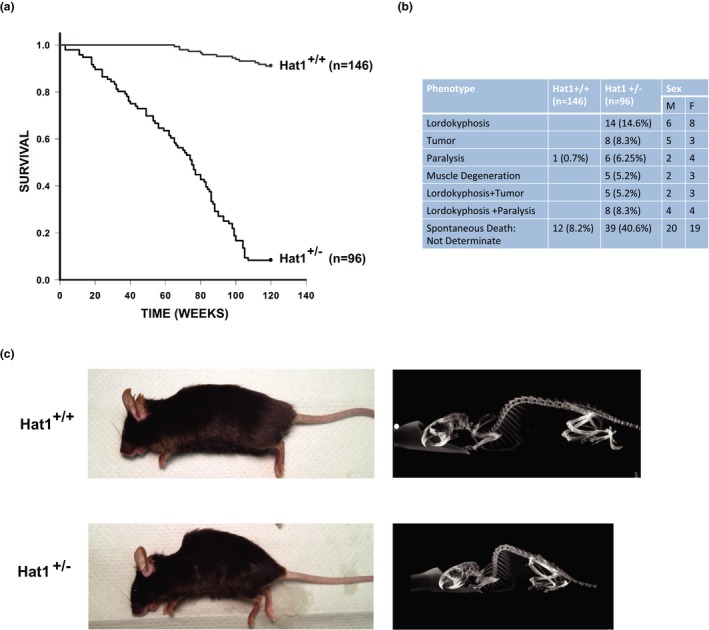
Haploinsufficiency of Hat1 reduces lifespan. (a) Kaplan–Meier survival curves of Hat1^+/+^ and Hat1^+/−^ mice. The percentages of survival are plotted as a function of age in weeks. Animals were monitored for tumors, morbidity or spontaneous death over a period of 120 weeks. All mice were of C57Bl6J background. The survival curves of the Hat1^+/+^ and Hat1^+/−^ mice were significant (*p* < 0.001). Survival curves were analyzed with GraphPad prism software using log‐rank test to determine statistically significant difference between survival curves of the two groups. Mean survival time for Hat1^−/−^ mice was 69.1 weeks. (b) Table shows pathologies observed at time of death. Hat1^+/−^ Total 96 (88 dead and 8 alive). Hat1^+/+^: Total 146 (13 dead and 133 alive). (c) Left, photographic image of 54‐week‐old Hat1^+/+^ and Hat1^+/−^ mice. Right, the same mice imaged using Small Animal Radiation Research Platform by Xstrahl, Hat1^+/+^, and Hat1^+/−^ mice cropped protocol entails: 180 projections at 60kV0.8mA fine focus w/0.5Al filter for 1.2cGy

Mice with a complete loss of Hat1 are not viable (Nagarajan et al., 2013). To identify potential physiological functions of Hat1, we characterized the effect of the loss of one copy of the HAT1 gene. We generated a total of 495 offspring from the 83 different intercrosses of Hat1^+/−^ animals. We obtained 312 heterozygotes and 183 wild‐type animals. Of these, 146 Hat1^+/+^ and 96 Hat1^+/−^ animals were used to determine the consequences of heterozygosity at the Hat1 locus. The animals were monitored for a period of 120 weeks. Over this period, 13 of the 146 Hat1^+/+^ animals died (9%), while 88 of the 96 Hat1^+/−^ animals died (92%). Analysis of survival curves indicated that the average lifespan of the Hat1^+/−^ mice was 69.1 ± 3.2 (s.e) weeks (Figure 1a). Mice that were found dead or were euthanized due to poor body score were assessed by overall body condition and by examination of major internal organs. Approximately 40% of the Hat1^+/−^ animals that died spontaneously could not be analyzed due to tissue lysis. Figure 1b lists the spectrum of phenotypes observed at the time of death. The most common phenotypes observed were lordokyphosis (Figure 1c), hindlimb paralysis, tumors, and muscle atrophy. Tumors were observed primarily in the liver, spleen, and kidney. The variety of abnormalities observed in Hat1^+/−^ animals suggested that they were not succumbing to a single disease state but was consistent with the premature appearance of phenotypes associated with aging.

### 2.2 Hat1 expression decreases during normal aging

**Figure 2:**
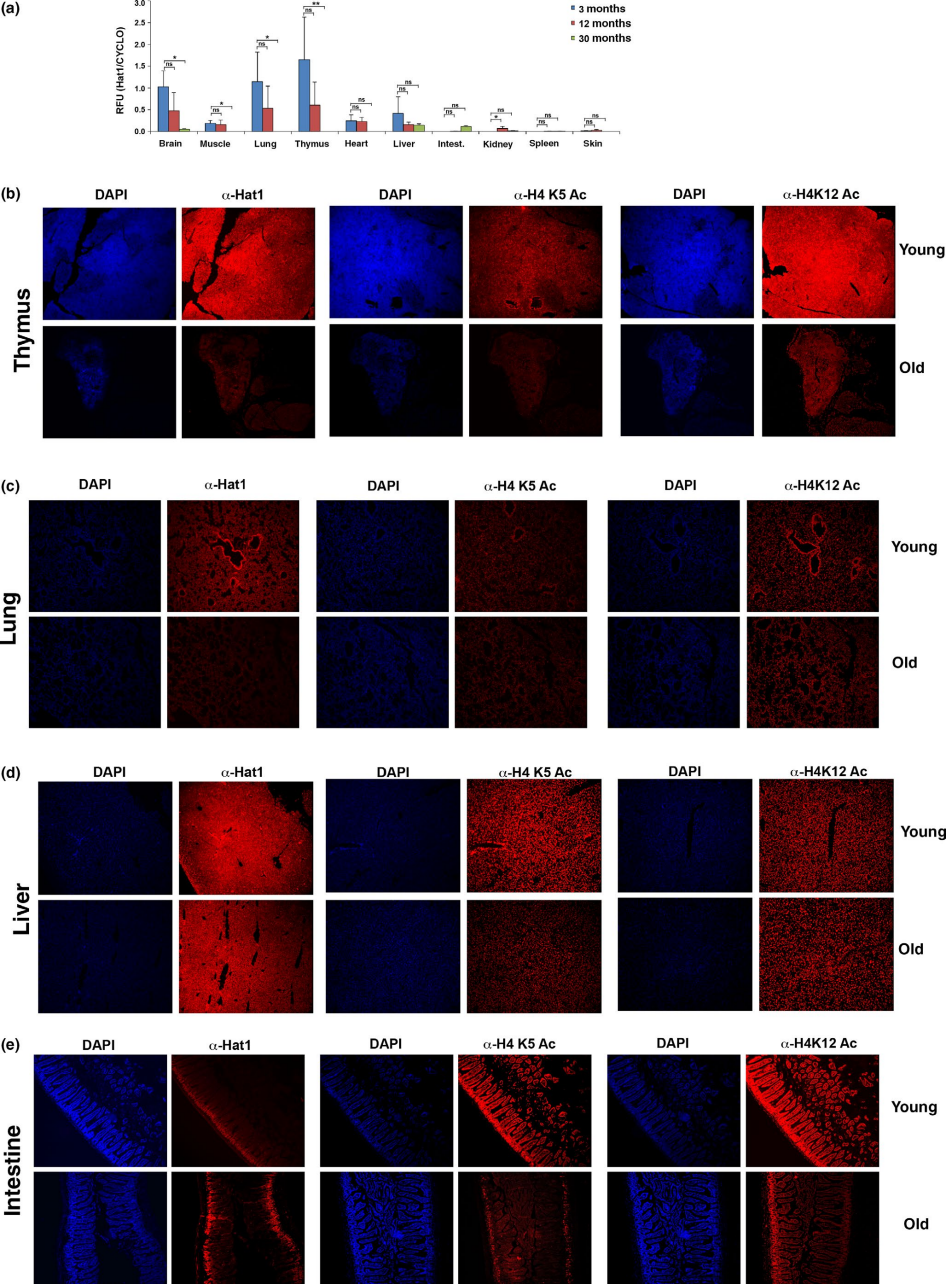
Tissue‐specific decreases in Hat1 expression occur during aging. (a) Hat1 mRNA levels were determined in the indicated tissues by digital droplet PCR. Tissues were harvested from Hat1^+/+^ animals sacrificed at 3, 12, or 30 months of age, as indicated (*n* = 3 animals per time point; ns = not significant; *p < 0.05; **p < 0.01). Levels of Hat1 mRNA are plotted relative to cyclophilin. (b–e) The indicated tissues were isolated from young (3 months) and old (31 months) mice. Tissue sections were stained with DAPI and with antibodies against Hat1, histone H4 lysine 5 acetylation, and histone H4 lysine 12 acetylation, as indicated. Images were taken at 10× magnification

To determine whether the expression of Hat1 was linked to the normal process of aging, Hat1^+/+^ mice were sacrificed at 3, 12, and 30 months of age (*n* = 3 animals at each time point). Hat1 mRNA levels were measured from several tissues at each of the time points. As seen in Figure 2a, Hat1 mRNA was variable but easily detectable in many tissues at 3 and 12 months of age. There was a significant decrease in Hat1 mRNA levels in thymus, lung, muscle, and brain at 30 months of age.

We determined the levels of Hat1 protein by immunofluorescence staining of sections of several different tissues from young and old Hat1^+/+^ mice (3 months and 31 months, respectively). We also analyzed the levels of histone H4 lysine 5 and lysine 12 acetylation, the primary known targets of Hat1, to determine whether they correlated with Hat1 protein levels. In general, an age‐dependent loss of Hat1 was observed that largely mirrored the results seen for Hat1 mRNA expression. There was Hat1 protein in most cells of the thymus in 3‐month‐old mice, while the number of Hat1‐positive cells decreased dramatically in 30‐month‐old mice (Figure 2b). The levels of histone H4 lysine 5 and lysine 12 acetylation closely mirrored that of Hat1, suggesting that Hat1 may be the primary enzyme responsible for these modifications in the thymus.

In the lung of young animals, Hat1 protein was present in only scattered nuclei with the exception of cells surrounding the alveoli, where most of the cells showed a high level of Hat1 protein (Figure 2c). Consistent with the Hat1 mRNA analysis, Hat1 protein was largely absent in the lung of old mice. The patterns of H4 lysine 5 and lysine 12 acetylation were not identical to the pattern of Hat1. H4 lysine 5 and lysine 12 acetylation was more widespread throughout the lung of young mice than Hat1. However, the levels of H4 lysine 5 and lysine 12 acetylation were also highest in the cells immediately surrounding the alveoli. Interestingly, in old mice, while much of the H4 lysine 5 and lysine 12 acetylation remained stable, the alveoli‐proximal staining was significantly reduced.

Hat1 protein was expressed in cells throughout the liver, and this expression was largely stable with age (Figure 2d). Histone H4 lysine 5 and lysine 12 acetylation was also widespread throughout the liver and was largely stable with age.

Interestingly, in the intestine, Hat1 protein was found specifically in cells of the crypt where intestinal stem cells are located (Figure 2e). The presence of Hat1 protein in these cells was not altered by age. The pattern of H4 lysine 5 and lysine 12 acetylation was complex. In young mice, both sites of acetylation were found evenly throughout all cells of the intestine. In old mice, the level of acetylation of both sites decreased dramatically and became predominantly localized to cells of the crypt.

An identical analysis was performed with tissues from young and old Hat1^+/−^ mice, 3 months and 24 months, respectively (we were not able to obtain Hat^+/−^mice older than 24 months). The results in the Hat1^+/−^ mice were similar to the Hat1^+/+^ except that the overall level of Hat1 expression in heterozygous mice was typically lower than in the wild‐type mice (Figure S1). Immunoblot analysis of Hat1, H4 lysine 5 acetylation, and H4 lysine 12 acetylation levels in these tissues in young and old mice was also performed (Figure S2). The immunoblot analyses largely mirror the results observed in the immunofluorescence images. Taken together, the results indicate that Hat1 protein expression is highly tissue‐specific and that normal aging can be accompanied by a marked loss of Hat1 mRNA and protein from some tissues. In addition, the acetylation state of H4 lysine 5 and lysine 12 acetylation can also vary with age but does not always correlate with Hat1 expression.

### 2.3 ***Hat1*** ^+/−^ ***animals exhibit aging‐related phenotypes***

**Figure 3:**
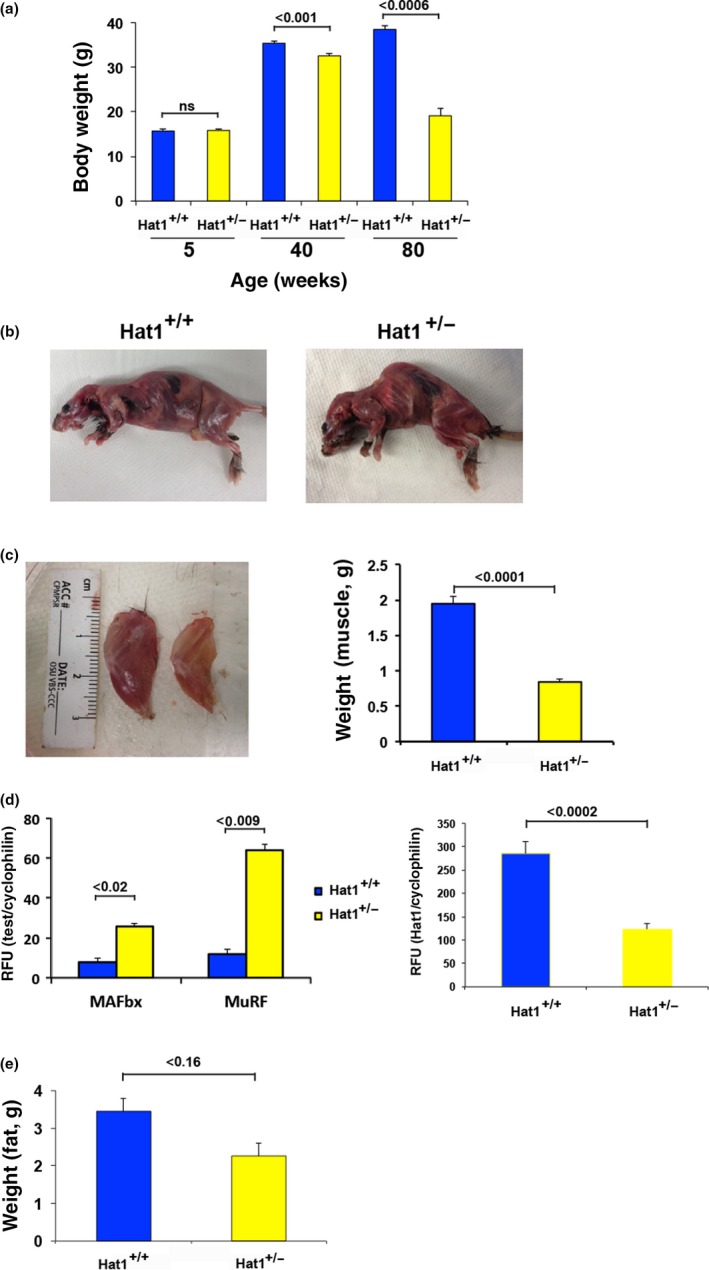
Haploinsufficiency of Hat1 results in decreased body and muscle mass. (a) Total body weight of Hat1^+/+^ and Hat1^+/−^ mice at the indicated ages (*n* = 6 for each genotype). (b) 54‐week‐old Hat1^+/+^ and Hat1^+/−^ skinned animals. (c) (Left Panel) Quadriceps muscles from 54‐week‐old Hat1^+/+^ (left) and Hat1^+/−^ (right) mice. (Right Panel) Weight of quadriceps muscle isolated from Hat1^+/+^ and Hat1^+/−^ mice (*n* = 6 for each genotype). (d) (Left Panel) Abundance of muscle atrophy markers MAFbx and MuRF1 in Hat1^+/+^ and Hat1^+/−^ muscle tissue assayed by ddPCR (*n* = 8 for each genotype). (Right Panel) Abundance of Hat1 mRNA in Hat1^+/+^ and Hat1^+/−^ muscle tissue (*n* = 8 for each genotype). (e) Total body fat in Hat1^+/+^ and Hat1^+/−^ mice as measured by EchoMRI (*n* = 8 for each genotype)

Loss of body weight can occur as mice reach old age (Alhurani et al., 2016; Goodrick, Ingram, Reynolds, Freeman, & Cider, 1990; Samorajski et al., 1985). Hat1^+/−^ mice displayed a reduced size at birth that became less pronounced by 5 weeks of age (Nagarajan et al., 2013). However, after 40 weeks, the Hat1^+/−^ mice began to exhibit weight loss and animals that survived to 80 weeks had a pronounced decrease in body weight (Figure 3a). Removal of the skin from Hat1^+/+^ and Hat1^+/−^ mice indicated that the Hat1 heterozygous animals appeared to have reductions in both muscle mass and body fat (Figure 3b). Quadriceps muscles from age‐matched Hat1^+/+^ and Hat1^+/−^ mice (54 weeks) were isolated and weighed. It was clear that Hat1^+/−^ mice had a significant loss of muscle mass (Figure 3c). To determine whether the loss of muscle mass in the Hat1^+/−^ animals was a result of muscle atrophy, we measured the transcript levels of MAFbx and MuRF1, genes that are upregulated in atrophying muscle tissue (Bodine et al., 2001). Indeed, there was a significant increase in the expression of both genes in the quadriceps muscle of Hat1^+/−^ animals (Figure 3d). As expected, there was a twofold decrease in Hat1 expression in the Hat1^+/−^ mice in these muscle tissues relative to the wild‐type animals (Figure 3d). The level of body fat in the Hat1^+/+^ and Hat1^+/−^ mice was analyzed using EchoMRI. As seen in Figure 3e, the results of this analysis, while not reaching statistical significance, suggested a trend toward decreased total body fat in the Hat1^+/−^ mice. Hence, Hat1^+/−^ mice lose muscle and fat mass consistent with the early onset of aging.

**Figure 4:**
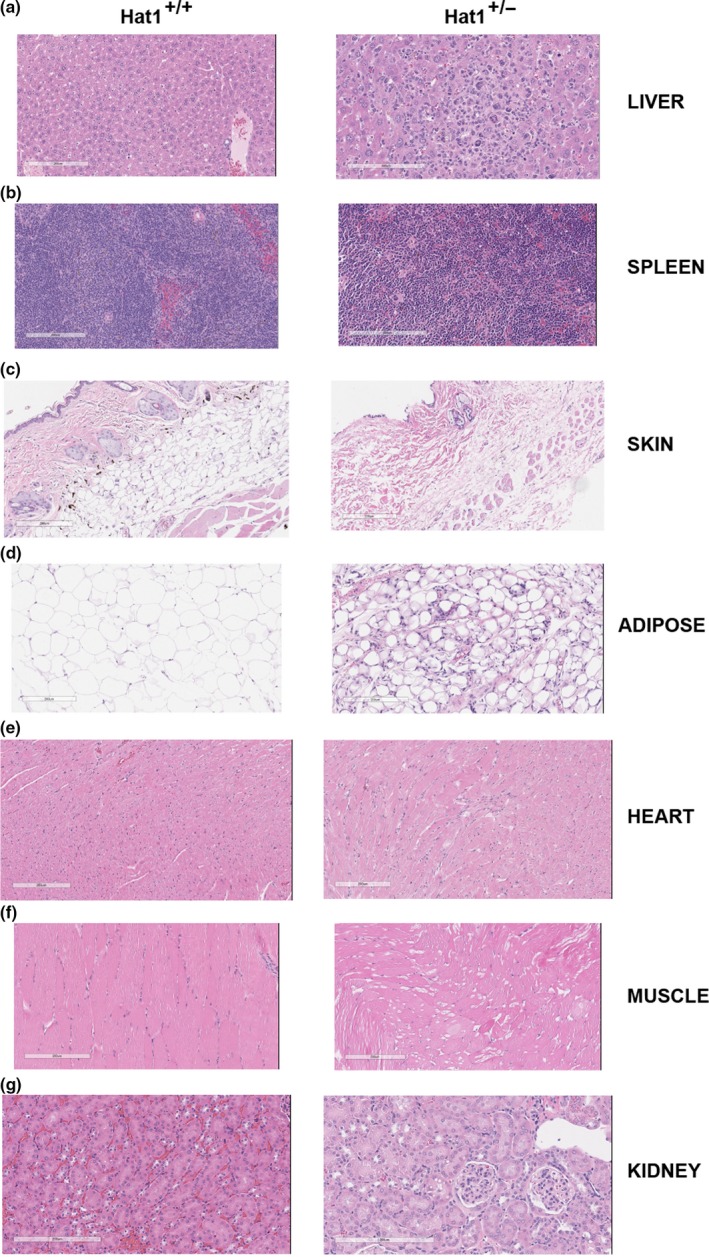
Hat1^+/−^ mice exhibit multi‐organ abnormalities. H&E‐stained samples from Hat1^+/+^ and Hat1^+/−^ were visualized under a microscope, and 200 μM images were taken. Samples were isolated from (a) liver, (b) spleen, (c) skin, (d) adipose, (e) heart, (f) muscle, and (g) kidney

To obtain a more complete view of the effect of Hat1 haploinsufficiency in mice, age‐matched Hat1^+/+^ and Hat1^+/−^ mice were randomly selected and sacrificed. The mice were between 54 and 80 weeks of age, which brackets the average lifespan of the Hat1^+/−^ mice. Tissue samples were isolated from a variety of organs, and an independent pathologist, blinded to the genotype of the tissue samples, performed a histological analysis. Table 1 summarizes the results of these analyses, and representative images are shown in Figure 4. For the Hat1^+/+^ tissues, there were no significant findings in any of the tissue samples with the exception of extramedullary hematopoiesis in two of the liver and spleen samples. The most striking finding in the liver and spleen of Hat1^+/−^ mice was the presence of histiocytic sarcoma in a high percentage of these animals (50% and 33%, respectively) (Table 1, Figure 4a,b). Interestingly, histiocytic sarcoma is a malignancy typically found in old mice (Blackwell, Bucci, Hart, & Turturro, 1995; Lacroix‐Triki et al., 2003).

**Table 1:** Pathological comparison of Hat1^+/+^ and Hat1^+/−^ mice

Tissue	Hat1^+/+^	Hat1^+/−^
Liver	2‐EMH (*n* = 12)	6‐histiocytic sarcoma; 4‐EMH; 2‐leukemia (*n* = 12)
Spleen	2‐EMH (*n* = 12)	4‐histiocytic sarcoma; 5‐EMH (*n* = 12)
Skin	No significant findings (*n* = 8)	8‐lack of well‐differentiated subcutaneous fat tissue (*n* = 8)
Adipose tissue	No significant findings (*n* = 3)	1‐fat depletion (*n* = 3)
Heart	No significant findings (*n* = 3)	3‐cardiomyocyte/intraventricular septum degeneration (*n* = 3)
Muscle	No significant findings (*n* = 3)	2‐moderate vacuolation (degeneration) (*n* = 3)
Kidney	No significant findings (*n* = 3)	2‐diffused, low number of tumor cells, leukemia (*n* = 3)

Abbreviation: EMH, Extramedullary hematopoiesis.

Comparison of skin from Hat1^+/+^ and Hat1^+/−^ animals revealed that the Hat1^+/−^ mice had a nearly complete lack of subcutaneous fat (Table 1, Figure 4c). There were also differences in visceral fat in the Hat1^+/−^ animals. The adipocytes from Hat1^+/−^ mice were much smaller, indicative of fat depletion in these cells (Table 1, Figure 4d). This dramatic loss of fat is another characteristic that links Hat1 loss with early‐onset aging.

The histological analyses also indicated the presence of muscle atrophy in the Hat1^+/−^ animals (Table 1, Figure 4e,f). In Hat1^+/−^ skeletal muscle, atrophy was indicated by vacuolation. In one of three Hat1^+/−^ heart samples examined, there was distinct evidence of intraventricular septum degeneration. Finally, two of the three kidney samples examined showed the presence of tumor cells, consistent with our frequent observation of tumors on the kidneys of the Hat1^+/−^ animals that spontaneously died.

### 2.4 Hat1 haploinsufficiency causes cellular senescence

**Figure 5:**
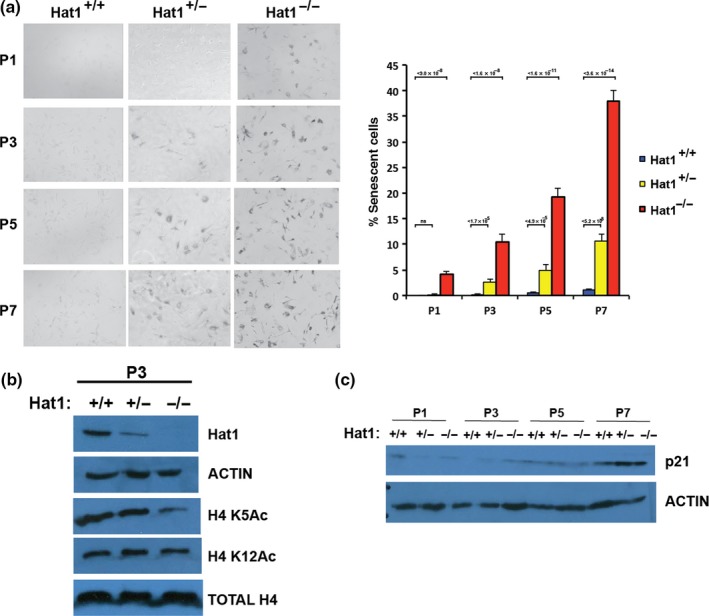
Loss of Hat1 results in cellular senescence. (a) (Left) MEFs isolated from mice of the indicated genotype were grown on agarose plates and stained for β‐galactosidase. Cells were plated following the indicated number of passages (P1 to P7). (Right) The percentage of senescent cells was quantitated following β‐galactosidase staining (*n* = 3). (b) Extracts from passage 3 MEFs of the indicated genotype were analyzed for the presence of proteins indicated on the right by Western blot analysis. (c) The levels of p21 in Hat1^+/+^, Hat1^+/−^, and Hat1^−/−^ MEFs at the indicated passage number were determined by Western blot analysis of whole‐cell extracts

Hat1^+/−^ mice displayed a range of phenotypes, suggesting that a decrease in Hat1 copy number results in an early onset of aging. To determine whether Hat1 influences aging‐related processes at the cellular level, we analyzed mouse embryonic fibroblast (MEF) cell lines derived from Hat1^+/+^, Hat1^+/−^, and Hat1^−/−^ embryos. Aging is often associated with an increase in cellular senescence. To determine the effect of Hat1 on senescence, primary MEFs at passages 1, 3, 5, and 7 were grown and then stained for β‐galactosidase (β‐gal) activity, a marker of senescent cells. As expected, the level of senescence increased with increasing passage number regardless of Hat1 status (Figure 5a). However, the level of senescence was clearly influenced by Hat1 copy number. Hat1^+/+^ cells displayed low levels of β‐gal staining at each passage. Hat1^−/−^ cells had significant levels of senescence, even at passage 1 (~5%), which increased to ~35% by passage 7. At each passage, Hat1^+/−^ cells displayed an intermediate level of senescence. To determine whether the levels of Hat1, H4 lysine 5 acetylation, and H4 lysine 12 acetylation correlated with the onset of senescence, we probed extracts from passage 3 cells for these proteins (Figure 5b). As expected, there is a significant decrease in Hat1 protein in the Hat1^+/−^ cells and it is completely absent in the Hat1^−/−^ cells. As seen in the tissue sections, the levels of H4 lysine 5 and lysine 12 acetylation do not necessarily change together and they are not entirely dependent on Hat1. H4 lysine 5 acetylation decreases in parallel with Hat 1 levels, but H4 lysine 12 acetylation is relatively unchanged despite the decrease in Hat1. Increased levels of p21 are used as a marker of senescent cells. Consistent with the β‐gal staining, there was a similar pattern of increase in the levels of the p21 with increasing passage number (Figure 5c). These data suggest that cellular senescence is sensitive to the amount of Hat1 in the cell and is consistent with the early onset of aging seen in Hat1^+/−^ animals.

### 2.5 Loss of Hat1 increases DNA damage and oxidative stress

**Figure 6:**
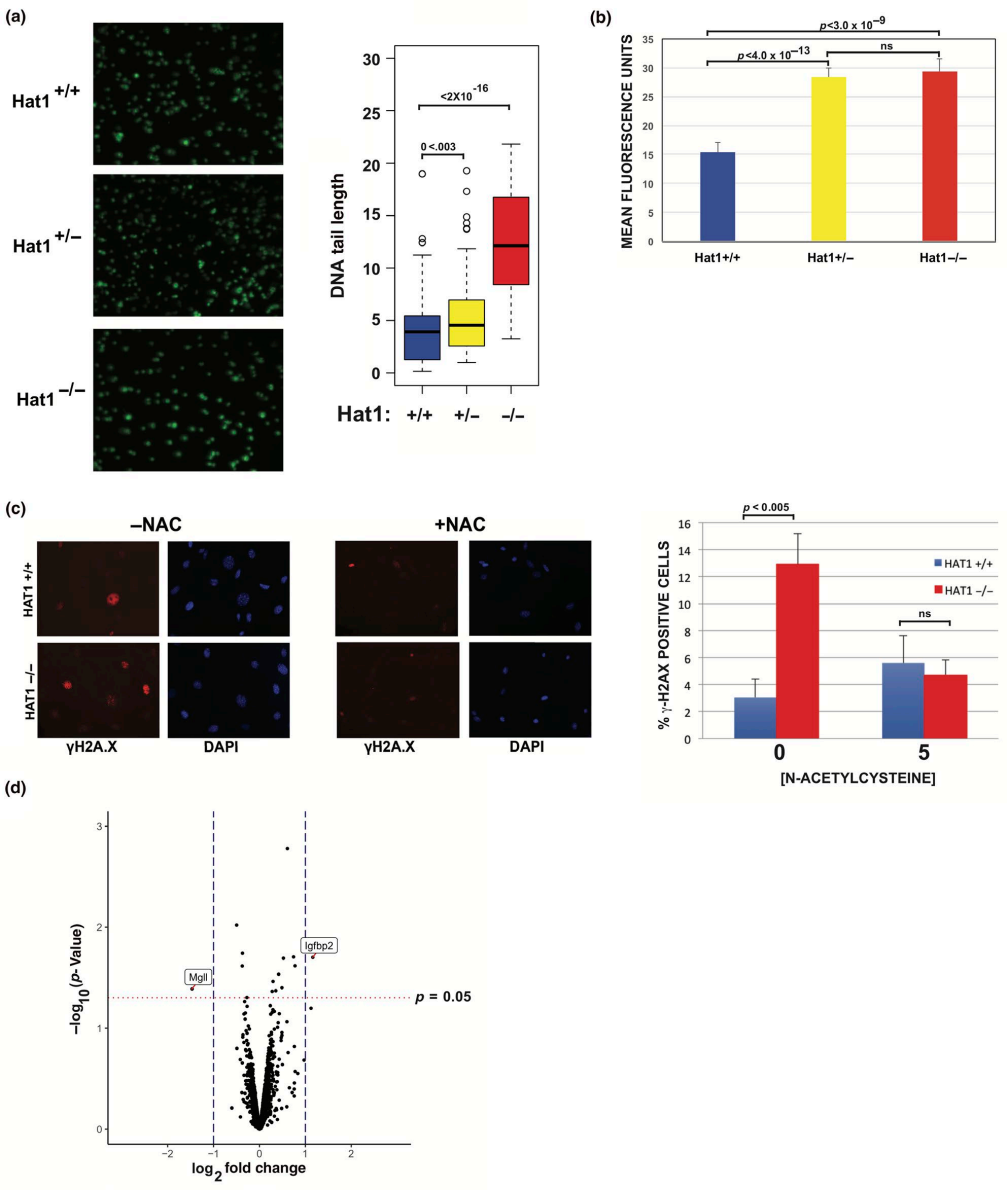
Loss of Hat1 results in increased levels of DNA double‐strand breaks and reactive oxygen species. (a) Endogenous DNA damage measured by comet assay analysis using passage 3 Hat1^+/+^, Hat1^+/−^, and Hat1^−/−^ MEFs (*n* = 3). (b) Reactive oxygen species were assayed by staining cells with DCFDA and measuring fluorescence intensity. (c) Cells of the indicated genotype were grown with or without 5 mM N‐acetylcysteine (NAC) for 48 hr before fixation and staining with anti‐ γ‐H2A.X antibody. Histogram shows percentage of γ‐H2A.X‐positive cells (*n* = 3). (d) Transcript levels, determined by RNA‐Seq, were compared between primary Hat1^+/+^ and Hat1^+/−^ MEFs. Results are shown as a volcano plot. Vertical dashed lines indicate a log_2_ fold change of + or – 1. Horizontal red dotted line indicates a *p* value of 0.05. Identity of transcripts that increase or decrease by at least ± log_2_ = 1 at a *p* value less than 0.05 is indicated

The accumulation of DNA damage is clearly an important driver of the aging process. In addition, defective DNA damage repair is an evolutionarily conserved phenotype observed in Hat1‐depleted cells in a wide variety of organisms (Barman et al., 2006; Benson et al., 2007; Qin & Parthun, 2002; Tscherner et al., 2012; Yang et al., 2013), including Hat1^−/−^ MEFs, which have been shown to exhibit increased levels of γ‐H2AX staining and genome instability (Nagarajan et al., 2013). Therefore, we investigated whether Hat1^+/−^ cells also displayed increased levels of DNA damage. Using comet assays to detect DNA double‐strand breaks, we found that Hat1^+/−^ cells showed a modest, but statistically significant increase in DNA damage, contrasting with the pronounced increase in DNA double‐strand breaks observed in the Hat1^−/−^ MEFs (Figure 6a).

Another important driver of aging, as well as spontaneous DNA damage, is oxidative stress. To determine whether loss of Hat1 influenced oxidative stress, we measured the levels of reactive oxygen species (ROS) in Hat1^+/+^, Hat1^+/−^, and Hat1^−/−^ MEFs by following the oxidation of 2',7'‐dichlorofluorescein diacetate (DCFDA) using fluorescence microscopy and quantifying the mean fluorescence intensity (Figure 6b). Surprisingly, both the Hat1^+/−^ and Hat1^−/−^ cells showed an approximately twofold increase in basal levels of ROS compared with Hat1^+/+^ cells.

To determine whether the high levels of ROS produced in the absence of Hat1 are responsible for the elevated levels of DNA damage observed in Hat1 mutants, we incubated the Hat1^+/+^ and Hat1^−/−^ MEFs with N‐acetyl‐cysteine (NAC), an acetylated amino acid that has been shown to minimize oxidative stress and its deleterious effects on the cell. After incubation with the antioxidant, DNA damage was measured by the presence of γ‐H2A.X foci. Analysis of at least 100 Hat1^+/+^ or Hat1^−/− ^cells showed that, as previously described, Hat1^−/−^ MEFs exhibit an approximately fivefold increase in γ‐H2A.X‐positive cells relative to Hat1^+/+^ cells in the absence of NAC (Nagarajan et al., 2013) (Figure 6c). Interestingly, when incubated with NAC, Hat1^+/+^ and Hat1^−/−^ cells had similar levels of γ‐H2X‐positive cells (Figure 6c). These results suggest that increased levels of oxidative stress are a key contributor to the increased levels of DNA damage seen in Hat1 mutants.

To determine whether the presence of DNA damage correlated with the loss of Hat1 during aging in vivo, tissue sections from young and old Hat1^+/+^ and Hat1^+/−^ mice were stained for the presence of Hat1 and γ‐H2AX (Figures S3 and S4). As expected, there was an age‐dependent increase in γ‐H2AX‐staining thymus, lung, and liver tissues. There were similar levels of γ‐H2AX staining in the young and old intestine. Interestingly, there was little colocalization between Hat1 and γ‐H2AX, suggesting that loss of Hat1 leads to increased DNA damage in vivo.

To comprehensively assess whether Hat1 heterozygosity results in alterations in gene expression that might be linked to the observed cellular defects, we performed RNA‐Seq analysis. Transcript levels were compared between primary Hat1^+/+^ and Hat1^+/−^ MEFs at passage 3. As described above, these Hat1^+/−^ cells had increased levels of senescence and elevated ROS. As seen in Figure 6d, there was very little change in gene expression in the Hat1^+/−^ cells. There is one gene, Igfrbp1, whose expression increased by greater than twofold, and there is one gene, Mgll, whose expression decreased by greater than twofold. Alterations in the expression of these genes are unlikely to result in increased cellular senescence or ROS. Hence, large‐scale changes in gene expression do not result from haploinsufficiency of Hat1 in MEFs.

### 2.6 Hat1 is required for proper mitochondrial function

**Figure 7:**
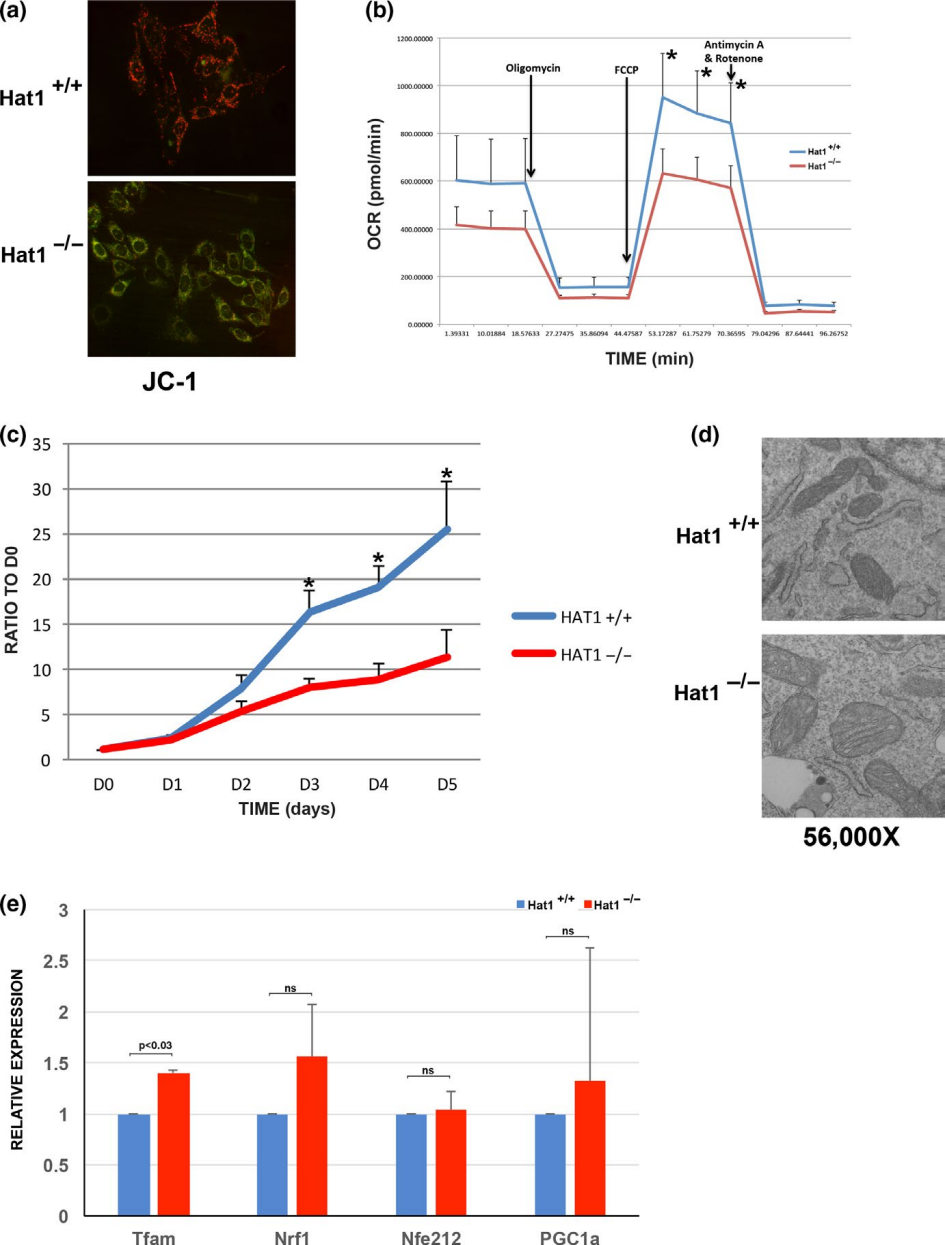
Loss of Hat1 leads to mitochondrial dysfunction. (a) MEFs of the indicated genotype were treated with vehicle or CCCP 30 min before staining with JC1 and imaging. (b) Mitochondrial stress profile of Hat1^+/+^ and Hat1^−/−^ MEFs. Arrows indicate the time points where the electron transport chain inhibitors were injected (*n* = 3). The * indicates *p* < 0.03. (c) Hat1^+/+^ and Hat1^−/−^ MEFs were switched from media containing glucose to media containing galactose on day 0 (D0) (*n* = 3). Cell numbers were measured every 24 hr and plotted relative to the cell number at day 0. The * indicates *p* < 0.02. (d) Electron microscopic images of mitochondria from Hat1^+/+^ and Hat1^−/−^ MEFs. (e) The abundance of the indicated mRNAs in Hat1^+/+^ and Hat1^−/−^ MEFs was measured by real‐time PCR (*n* = 3)

Increased ROS production is typically a symptom of mitochondrial dysfunction. To determine whether Hat1 has an unidentified role in mitochondrial function, we compared various aspects of mitochondrial function between Hat1^+/+^ and Hat1^−/−^ MEFs as subtle Hat1‐dependent alterations in mitochondrial function are more likely to be apparent in the complete absence of Hat1. Disruption of active mitochondria induces alterations in the membrane potential used during the respiration process to produce energy. Fluorescence microscopy after JC‐1 staining was used to measure mitochondrial membrane potential in Hat1^+/+^ and Hat1^−/−^ MEFs. Polarized mitochondria retain highly concentrated JC‐1 aggregates that emit red fluorescence, while in depolarized mitochondria, JC‐1 leaks out to the cytosol forming monomers that emit green fluorescence. Hence, a shift from red to green indicates loss of membrane potential. Intriguingly, while Hat1^+/+^ MEFs were predominantly red, Hat1^−/−^ MEFs displayed largely green fluorescence, indicating that loss of Hat1 induced mitochondrial depolarization (Figure 7a).

Loss of mitochondrial membrane potential is associated with uncoupled oxidative phosphorylation that arises from either defect in the ATP synthase or to increased permeability of the mitochondrial inner membrane (Sack, 2006). To determine whether the loss of mitochondrial membrane potential in the absence of Hat1‐induced defects in mitochondrial respiration, we evaluated the metabolic profiles of Hat1^+/+^ and Hat1^−/−^ MEFs using the Seahorse® system. We analyzed oxygen consumption rate (OCR) as a measure of mitochondrial respiration and tested different parameters of mitochondrial function by using inhibitors for the different complexes of the electron transport chain. As seen in Figure 7b, Hat1^−/−^ cells exhibited a lower basal respiration rate than Hat1^+/+^ cells and a decreased maximal respiratory capacity. These findings suggest that loss of Hat1 impairs mitochondrial respiration.

After establishing that key parameters of the mitochondrial metabolism are impaired in Hat1^−/−^ cells, we evaluated the cellular capacity to respond to nutrient stress. Under normal culture conditions, mammalian cells do not rely heavily on mitochondria for growth, as they are able to metabolize the glucose contained in the media through glycolysis. However, growth on alternative carbon sources, such as galactose, requires functional mitochondria for proliferation. Cells grown in galactose increase their respiration rate in order to maintain adequate ATP levels. Since the conversion of galactose to glucose yields no net ATP, cells are forced to rely only on mitochondrial respiration to generate the required ATP for optimal growth (Haigis, Deng, Finley, Kim, & Gius, 2012). To confirm a role for Hat1 in mitochondrial function, Hat1^+/+^ and Hat1^−/−^ cells were incubated in glucose‐free media supplemented with galactose and time courses for cell viability were generated over a five‐day period (Figure 7c). The difference between Hat1^+/+^ and Hat1^−/−^ cells was striking. Wild‐type cells exhibited a typical exponential growth curve achieving more than a 20‐fold increase in cell proliferation by day five. In contrast, cells lacking Hat1 grew poorly on galactose. These results show that Hat1 is required for the function of mitochondria in mammalian cells.

We next examined whether loss of Hat1 resulted in morphological defects to mitochondria. Hat1^+/+^ and Hat1^−/−^ MEFs were examined by transmission electron microscopy. As seen in Figure 7d, mitochondria from Hat1^+/+^ cells had a normal appearance. However, mitochondria in the Hat1^−/−^ cells were clearly defective. The mitochondria are enlarged, with disorganized cristae that have an open structure. These morphological defects are consistent with the mitochondrial functional defects observed in the Hat1^−/−^ cells.

A recent report linked Hat1 to mitochondrial function in HUVEC cells through the transcriptional regulation of genes important for mitochondrial function and biogenesis. In this study, AMPK was found to phosphorylate and activate Hat1 activity. It was then proposed that activation of Hat1 led to the acetylation and induction of several genes important for mitochondrial function, including Tfam, Nrf1, Nrfe2L2 (Nrf2), and PGC‐1αΔ (Marin et al., 2017). To determine whether the role of Hat1 in mitochondrial function in MEFs is through a similar mechanism, we measured the abundance of these transcripts in Hat1^+/+^ and Hat1^−/−^ cells. However, as seen in Figure 7e, the expression of these genes was not dependent on Hat1. Together, our results indicate that Hat1 is not required for the expression of genes that regulate mitochondrial function and suggest a more direct role for Hat1 in mitochondrial function.

## 3 DISCUSSION

Our results indicate that Hat1^+/−^ mice are viable but have a much shorter lifespan than Hat1^+/+^ mice. The Hat1^+/−^ mice do not develop a uniform disease state prior to their death. Rather, they develop an array of phenotypes that are consistent with the early onset of aging. These phenotypes include lordokyphosis, muscle atrophy, loss of subcutaneous fat, and the development of malignancies.

While there is considerable variation between tissue types, there is a clear trend toward decreased expression of Hat1 as mice age. This is apparent at both the mRNA and protein levels. While it is not clear whether the normal process of aging requires a loss of Hat1, our observation that a decrease in Hat1 copy number leads to the early onset of aging suggests that Hat1 expression may be an important factor in mammalian aging.

Early‐onset aging is often associated with defects in specific cellular processes, which include DNA damage repair, cellular senescence, epigenetic regulation of gene expression patterns, and mitochondrial function. Our results indicate that Hat1 may influence aging through any, or all, of these pathways.

A role for Hat1 in DNA damage repair is conserved in a wide array of eukaryotes. Deletion of the *HAT1* gene in *Saccharomyces cerevisiae* (in combination with specific mutations in histone H3) leads to specific defects in the recombinational repair of DNA double‐strand breaks (Qin & Parthun, 2002). Hat1p appears to function directly in the repair process as it is recruited to sites of double‐strand breaks, influences histone H4 acetylation near the break sites, and promotes chromatin reassembly following repair (Ge et al., 2011; Qin & Parthun, 2006). Similar results were observed in a human tissue culture model (Yang et al., 2013). Hat1‐dependent sensitivity to DNA damage has also been observed in *S. pombe*, chicken DT40 cells, and *C. albicans* (Barman et al., 2006; Benson et al., 2007; Tscherner et al., 2012).

Hat1 may influence the epigenetic regulation of gene expression in a number of ways. First, the role of Hat1 in the acetylation of newly synthesized histone H4 during chromatin assembly may influence the reproduction of the normal epigenetic landscape following DNA replication. In the absence of Hat1, loss of acetylation on the newly synthesized H3/H4 tetramers may disrupt the mechanisms by which localized patterns of histone modification are transmitted to the newly synthesized histones (Agudelo Garcia et al., 2017). This model is consistent with the multiple developmental defects observed in Hat1^−/−^ mice (Nagarajan et al., 2013). Recent evidence suggests that Hat1 may play a more direct role in transcriptional regulation. In tumor‐associated Treg cells, Hat1 was found to physically associate with the transcription factor FoxP3. This FoxP3 complex could be localized to the IL‐10 promoter region through interaction with Stat3, resulting in Hat1‐dependent modification of IL‐10 promoter chromatin and induction of IL‐10 expression (Hossain et al., 2013). Hat1 was also shown to be involved in transcriptional repression. Signaling by Toll‐like receptors or TNFα receptors can activate CamK2. It was found that activated CamK2 could phosphorylate Hat1. This phosphorylation stimulates Hat1 to acetylate the transcriptional repressor PLZF. Acetylation of PLZF triggers the formation of a transcriptional repressor complex that limits NF‐κB signaling (Sadler et al., 2015). Hence, Hat1 can have a range of effects on the regulation of transcription and can function through multiple mechanisms.

There are multiple mechanisms through which Hat1 may influence mitochondrial function. A previous report suggested that Hat1 was required for the expression of several nuclear genes important for mitochondrial biogenesis and function (Marin et al., 2017). However, this may be a cell type‐specific phenomenon, as we see no evidence for Hat1‐dependent expression of these genes in our Hat1^−/−^ MEFs, where clear defects in mitochondrial function are observed.

A more intriguing possibility is that Hat1 may affect mitochondrial function more directly through the acetylation of mitochondrial proteins. Acetylomic studies have shown that the majority of mitochondrial proteins are acetylated and that the acetylation state of mitochondrial proteins can be a key regulator of protein function (Baeza, Smallegan, & Denu, 2016; Wang et al., 2010; Zhao et al., 2010). There are a limited number of protein acetyltransferases in mammalian cells, and the majority of these enzymes are strictly localized to the nucleus. Hat1 is one of the few protein acetyltransferases that is not restricted to the nucleus. Whether Hat1 can localize to the mitochondria remains to be determined, but Hat1 may also influence mitochondrial protein acetylation through the acetylation of mitochondrial proteins in the cytoplasm prior to their import into the mitochondria.

Protein acetylation has been clearly linked to aging through the Sirtuin family of proteins. The Sirtuins are NAD^+^‐dependent protein deacetylases. A role for Sirtuins in regulating aging was first identified in *S. cerevisiae*, where overexpression of Sir2p increases lifespan and *sir2*Δ mutants show decreased lifespan (Kaeberlein et al., 1999). There are multiple Sirtuins in mammalian cells, several of which influence aging processes. For example, SirT1 is a nuclear deacetylase whose substrates include a number of proteins important in aging, such as p53, PGC1a, Nf‐kB, and fork head proteins (Haigis & Guarente, 2006). While knockout of SirT1 leads to neonatal lethality in mice, overexpression has an anti‐aging effect on a number of tissues (Alcendor et al., 2007; Kim et al., 2007; Leibiger & Berggren, 2006; Sommer et al., 2006; Wang et al., 2016). SirT3 is a mitochondrial protein deacetylase that is responsible for regulating the acetylation state of many mitochondrial proteins. Mutations in SirT3 in mice result in defects in many of the same processes implicated in aging, such as increased incidence of cancer, cardiovascular disease, metabolic syndrome, and neurodegenerative disease (McDonnell et al., 2015). Another Sirtuin, Sirt6, is also a nuclear protein deacetylase. Sirt6^−/−^ mice display rapid aging and typically die by one month of age (Mostoslavsky et al., 2006). The spectrum of phenotypes observed in Sirt6^−/−^ mice is strikingly similar to the phenotypes we observe in Hat1^+/−^, including lordokyphosis, loss of subcutaneous fat, and genome instability.

It seems counterintuitive that loss of Sirtuin protein deacetylases in mice would share aging‐related phenotypes with loss of the histone acetyltransferase Hat1. However, this has been observed in other systems. Deletion of *SIR2* in yeast results in loss of telomeric silencing (Grunstein, 1997). Similarly, deletion of *HAT1*, in combination with mutations in histone H3, results in loss of telomeric silencing (Kelly, Qin, Gottschling, & Parthun, 2000). Explanations for similar phenotypes resulting from loss of protein deacetylase and acetyltransferase activities include the possibility that Hat1 is a regulator of Sirtuin activity or that intact cycles of acetylation and deacetylation are necessary for the function of common targets of Hat1 and the Sirtuins.

Mice have served as an important mammalian model system for aging research (Harkema, Youssef, & Bruin, 2016; Koks et al., 2016; Quarrie & Riabowol, 2004; Yuan, Peters, & Paigen, 2011). There are a number of models that demonstrate an extremely rapid onset of aging and that model human diseases of aging. These include mutations in the LMNA gene that model Hutchinson‐Guilford Progeria (Osorio et al., 2011; Yang, Andres, Spielmann, Young, & Fong, 2008). Other mouse models of extreme aging include mutations in the Werner syndrome helicase (WRN) (Lebel & Leder, 1998). These models typically have a lifespan of 1 to 3 months. Other mouse models that have very short lifespans (2–3 months) include mutations in genes important for mitochondrial function (e.g., PolG), DNA damage repair (e.g., XPD, ERCC1), and immune function (e.g., IL‐10) (de Boer et al., 2002; Kuhn, Lohler, Rennick, Rajewsky, & Muller, 1993; Kujoth et al., 2005; Trifunovic et al., 2004; Walston et al., 2008; Weeda et al., 1997). There are relatively few mouse models of premature aging that have the type of intermediate phenotype seen with the Hat1^+/−^ animals. A particularly interesting example is mice heterozygous for p53. P53^+/−^ mice have a lifespan similar to that of Hat1^+/−^ mice and have a number of phenotypes in common. Interestingly, heterozygosity for p53 significantly increases the lifespan of some progeroid‐like mouse aging models and Sirt6^−/−^ mice (Ghosh et al., 2018). This suggests that p53 signaling can have a profound influence on the aging process.

Metformin may provide an intriguing link between Hat1 and aging. Metformin, which is used to lower blood sugar, has shown potential as an anti‐aging therapeutic. Metformin functions by inducing AMPK activity. While AMPK down‐regulates many factors involved in epigenetic regulation, it phosphorylates and activates Hat1 (Bridgeman, Ellison, Melton, Newsholme, & Mamotte, 2018). A primary target of Hat1 acetylation, histone H4 lysine 12, has also been linked to aging. Increased acetylation of H4 lysine 12 is associated with learning and memory in the hippocampus of mice. With age, the acetylation of H4 lysine 12 is lost along with the ability to form memories. Importantly, increasing H4 lysine 12 acetylation through treatment with HDAC inhibitors restored learning and memory in older mice (Peleg et al., 2010). These results suggest that modulation of Hat1‐dependent acetylation may be a therapeutic anti‐aging strategy.

## 4 EXPERIMENTAL PROCEDURES

### 4.1 Generation of Hat1 mutant mice

Hat1^+/−^ mice were generated as described previously (Nagarajan et al., 2013). Heterozygous mice were backcrossed to a C57BL6 background for more than ten generations. All mice were bred and housed in a pathogen‐free facility, and all studies were conducted in accordance with guidelines of the institutional animal care and use committee and university laboratory animal resources at The Ohio State University under protocol number 2007A0094.

### 4.2 Generation of primary mouse embryonic fibroblasts

Mouse embryonic fibroblasts (MEFs) derived from embryos obtained from crosses of Hat1^+/−^ males and females. Pregnant heterozygous female mice were euthanized at 13.5dpc, and the embryos were cultured to generate Hat1^+/+^, Hat1^+/−^, and Hat1^−/−^ fibroblasts. These MEFs were cultured in Dulbecco's modified Eagle's medium–high glucose (Sigma) supplemented with 10% fetal bovine serum (FBS) and 1X penicillin/streptomycin antibiotics. SV40 T immortalized MEFs (iMEFs) were derived from primary Hat1^+/+^ and Hat1^−/−^ embryonic day 13.5 embryos. To establish iMEFs, early passage cells were transformed with SV‐40T antigen containing plasmid pBSSVD2005 (ADDGENE). Early passage cells were seeded at 25% confluency in 6‐well plates and transfected with 2 μg of expression vector using Fugene reagent (Roche). Cells were harvested and seeded into 100‐mm dishes after 48 hr of transfection. The cells were split at 1 in 10 dilutions until passage 5.

### 4.3 Histology analyses

Mouse tissues were fixed in 10% formalin phosphate buffer, embedded in paraffin, sectioned, and stained with hematoxylin and eosin. Tissue sections were read by an independent pathologist (Histowiz, Inc), who was blinded to the groups being studied.

### 4.4 Body fat analysis

EchoMRI were used to measure body fat in live Hat1^+/+^ and Hat1^+/−^ mice. Mice were placed in a plastic cylinder without sedation or anesthetic agent. The cylinder was then inserted into a tubular space in the EchoMRI™ system. EchoMRI was performed at OSU small animal imaging core laboratory (SAIC).

### 4.5 Imaging

Hat1^+/+^ and Hat1^+/−^ mice skeletal images were taken using Small Animal Radiation Research Platform (SARRP) by Xstrahl. Mouse cropped protocol entails: 180 projections at 60 kV 0.8mA fine focus w/0.5 Al filter for 1.2cGy. Images were analyzed by Muriplan imaging software.

### 4.6 Comet assay

DNA double‐strand breaks in Hat1^+/+^, Hat1^+/−^, and Hat1^−/−^ mouse embryonic fibroblasts (MEFs) were detected by single cell gel electrophoresis by using a Comet Assay Kit (Trevigen) according to the manufacturer instructions. Briefly, MEFs were resuspended in ice‐cold PBS (Ca^2+^ and Mg^2+^ free) to a concentration of 1 × 10^5^ cells/ml. 10 μl of cells was mixed with 100 μl of warm low‐melting Agarose, and then, 50 μl was evenly spread onto comet slides. Slides were stored at 4°C in the dark for increasing gelling time to 30 min to improve adherence of samples on slides and transferred to prechilled lysis solution for 60 min at 4°C. Slides were transferred to alkali unwinding solution at room temperature for 60 min. in a dark chamber. Slides were transferred to electrophoresis system which contained prechilled alkaline electrophoresis solution and run at 1 V/cm, 300 mA for 45 min at 4°C. The slides were rinsed twice with sterile water for 5min. and washed in 70% ethanol for 5 min. Slides were stained with 100μl of SYBR Green I for 5–10 min in the dark, and slides were analyzed under Zeiss Axiophot fluorescence microscope. Images were taken using Metavue software version 6.3r2 software, and comet tails were analyzed using OpenComet by ImageJ. Dotplot was generated by SigmaPlot 12.0.

### 4.7 Senescence assay

Hat1^+/+^, Hat1^+/−^, and Hat1^−/−^ MEFs were seeded in 2‐well chamber slides (Lab‐Tek). MEFs were stained with β‐galactosidase staining kit according to the manufacturer's protocol (Invitrogen). Stained slides were visualized with a Nikon DIAPHOT 300 microscope, and images were captured using imaging software for microscopy SPOT 5.3. The percentage of senescent cells was calculated as the total number of β‐galactosidase staining cells divided by the total number of cells.

### 4.8 RNA extraction and droplet digital PCR

Total RNA from Hat1^+/+^ and Hat1^+/− ^muscle tissue was prepared using Trizol reagent (Invitrogen). 2 μg of RNA was used for cDNA synthesis reactions performed using High Capacity cDNA Reverse Transcription Kit (Applied Biosystems). Hat1 and muscle atrophy markers (MuRF1 and MAFbx) mRNA levels were determined using droplet digital PCR (ddPCR, Bio‐Rad) as per the manufacturer's instructions (Iyer, McGovern, Wise, Glass, & Burghes, 2014). Briefly, the sample was mixed with primers, probe, and the ddPCR supermix and nanodroplets were generated in the droplet reader (Bio‐Rad). After PCR, the fluorescence of the droplets was measured in the ddPCR droplet reader. The amplified PCR product in the droplets was analyzed by QuantaSoft analysis software, using Poisson statistics (Bio‐Rad). Technical replicates were performed, and relative fluorescence levels were determined by normalizing with cyclophilin mRNA*.* The primers and probes used for mRNA analyses were as follows: Hat1: Forward 5′‐*ctgagcaatacagaagctacag*‐, Reverse 5′‐*tctggtctcaggcatttcttc*‐, Probe‐FAM‐5′‐acaagaaaaagcagagggatcttgccaaga‐ZEN/IBFQ. MAFbx: Forward 5′‐*tccttatgcacactggtgca*, Reverse 5′‐*ctcagcctctgcatgatgttc*, Probe‐FAM‐5′‐*caacattaacatgtgggtgt*‐ZEN/IBFQ; *MuRF1*: Forward 5′‐*agctgagtaactgcatctccatgc*, Reverse 5′‐*ttctgctccaggatggcgta*, Probe‐FAM‐5′‐*cgagtgcagacgatca*‐ZEN/IBFQ; Cyclophilin: Forward 5′‐gtcaaccccaccgtgttctt, Reverse 5′‐ttggaactttgtctgcaaaca, Probe‐VIC‐5′cttgggccgcgtct‐MGB.

### 4.9 RNA‐seq analysis

For RNA sequencing analysis, total RNA was extracted from primary mouse embryonic fibroblasts using Total RNA purification plus kit (Norgen Biotek Corp.) according to the manufacturer's instructions. The RNA quality was tested using Agilent 2,100 Bioanalyzer, and RNA libraries were prepared using 200 ng total RNA as per manufacturer's instructions for the TruSeq RNA Sample Prep Kit (v2, Illumina). Concentration and size distribution of completed libraries was determined using an Agilent Bioanalyzer. Libraries were sequenced to a depth of 37–45 million pass filter clusters per sample following standard protocols using the Illumina cBot and cBot Paired‐end cluster kit (version 3). Flow cells were sequenced on an Illumina HiSeq 2000 using TruSeq. sequencing kit and HCS (v2.0.12) data collection software. Transcriptome measurements from Hat1^+/+^ and Hat1^+/−^ primary mouse embryonic fibroblasts were performed in biological triplicates. Statistical analysis was performed in R. Volcano plot was created using *ggplot2* (Wickham, 2009). For labeling purposes, transcripts were considered statistically significant if they exhibited greater than twofold change in abundance and a p value of less than 0.05.

### 4.10 Immunoblotting

Whole‐protein lysates from MEFs and mouse tissues were prepared by using RIPA buffer (Research product International—R26200—100 mM Tris‐HCl pH 7.4; 300 mM NaCl; 2% NP‐40; 1% sodium Deoxycholate; 0.2% SDS). For the analysis of senescent cells, two cell lines of each genotype were combined in order to obtain sufficient cells to analyze. Samples were separated in either 10% or 18% acrylamide gels and transferred to nitrocellulose blotting membrane (cat.# 10600004‐GE Healthcare Life Sciences). The membrane was blocked with 5% skim milk in TBS‐T (20 mM Tris‐HCl at pH 7.4, 150 mM NaCl, 0.1% Tween‐20) for 60 min at room temperature and then incubated with primary antibodies against Hat1 (Abcam, ab12163), beta‐Actin (Santa Cruz Biotech. SC47778), H4 (Abcam, ab10158), H3 (Abcam, ab1791), H4K5 (Abcam, ab51997), H4K12 (Abcam, ab46983), and P21 (Santa Cruz Biotech, sc‐6246) overnight at 4°C. HRP‐conjugated secondary antibodies and HyGLO quick spray chemiluminescent (cat.# E2400‐Denville Scientific Inc.) were used for detection.

### 4.11 Immunofluorescence (IF) on paraffin embedded mouse tissue sections

Mouse tissues were fixed in 10% buffered formalin phosphate solution (cat.# SF100‐4; Fisher Scientific) for 48 hr and transferred to 70% EtOH. Tissues were processed, embedded in paraffin, and cut in 8 micron thickness section on positively charged slides. Paraffin‐embedded tissues were deparaffinized using a xylene–ethanol series and subjected to antigen retrieval using Target Retrieval solution (cat.# S1700; Dako North America, Inc.) in a steamer for 60 min. The sections were then cooled to RT, blocked for 60 min. at RT in 1% Antibody dilution buffer (10% ADB in PBSpH7.4:3% BSA, 10% Goat serum, 0.05% Triton X‐100), and then incubated overnight with primary antibodies (Hat1; H4K5 and H4K12–Abcam; γ‐H2AX ab26350, Abcam) in antibody dilution buffer at 4°C. Next day, slides were washed three times in 1% ADB and incubated with Alexa Fluor^®^ 594‐conjugated secondary antibody (Jackson immunoresearch Lab. Inc) diluted with ADB in the dark for 60min. Following three times PBS washes, the slides were mounted in Vectashield mounting medium with DAPI (cat.# H1200, Vector Laboratories, Burlingame, CA). Photographs were taken by ANDOR microscope by Metamorph 64bit Software at The Ohio State University Neuroscience Microscopy Core.

### 4.12 Detection of reactive oxygen species

Cells were incubated with 25 μM DCFDA for 30 min at 37°C in the dark. ROS generation was assessed using fluorescence microscopy.

### 4.13 Determination of mitochondrial membrane potential

Cells were incubated with 1 μg/mL JC‐1 (Life Technologies) for 30 min at 37°C in the dark. Cells were then washed twice with PBS. Changes in mitochondrial membrane potential were determined using fluorescence microscopy.

### 4.14 Determination of cell viability

Cells were plated in a 96‐well plate. After 24 hr, cells were starved by changing to media containing 10 mM galactose. Cell viability was determined by MTS assay using the CellTiter 96 Aqueous One Solution according to the manufacturer's protocol (Promega, Madison, WI, USA). Absorbance was read at 490 nm.

### 4.15 Metabolic measurements

Oxygen consumption measurements were performed on the XF24 analyzer (Seahorse Bioscience) following the manufacturer's protocol. Briefly, cells were plated in 24‐well plates and incubated overnight at 37°C. The next day, cells were incubated in assay media for at least 1 hr before measurements. Wells containing cells were sequentially injected with 1 μM oligomycin, 2 μM FCCP, and 0.5 μM antimycin A and rotenone.

### 4.16 Statistical analysis

Mouse survival curve analysis was calculated by the Kaplan–Meier method, and differences were determined by the log‐rank test. All statistics were performed using sigmaplot 12.0. Results are presented as mean ± SE. Comparisons of experimental groups were carried out with Student's *t* test. Differences among multiple groups were analyzed by a one‐way ANOVA. *p* < 0.05 was considered to be significant when analyzing statistical differences between Hat1^+/+^, Hat1^+/−^, and Hat1^−/−^.

## CONFLICT OF INTEREST

None declared.

## AUTHOR CONTRIBUTIONS

P.N planned and executed experiments, analyzed data, and wrote the manuscript; P.A.A.G. planned and executed experiments, analyzed data, and wrote the manuscript. C.I. planned and executed experiments, analyzed data, and edited the manuscript. L.V.P. performed the analysis of the RNA‐Seq data. W.D.A. analyzed data and edited the manuscript. M.R.P. designed experiments, analyzed data, and wrote the manuscript.
