# Supplementary material for: Early‐onset aging and mitochondrial defects associated with loss of histone acetyltransferase 1 (Hat1)
Source: Aging Cell. 2019 Jul 10;18(5):e12992. doi: 10.1111/acel.12992 (PMC6718594; doi:10.1111/acel.12992)
Supplement: Supplementary file 1 [file ACEL-18-e12992-s001.pdf]

Supplemental Material

Contents:

Supplemental Table 1. Excel file containing analysis of RNA-Seq data from 3 $Hat1^{+/+}$ and 3 $Hat1^{+/-}$ cell lines.

Supplemental Figure 1. Tissue-specific decreases in Hat1 expression occur during aging in $Hat1^{+/-}$ mice. A-D. The indicated tissues were isolated from young (3 month) and old (24 month) $Hat1^{+/-}$ mice. Tissue sections were stained with DAPI and with antibodies against Hat1, histone H4 lysine 5 acetylation and histone H4 lysine 12 acetylation, as indicated. Images were taken at 10X magnification.

Supplemental Figure 2. Immunoblot analysis of Of Hat1, H4 lysine 5 and 12 acetylation in young and old mice. Lysates were generated from the indicated tissues from $Hat1^{+/+}$ (WT) and $Hat1^{+/-}$ (Het) mice. Tissues were harvested from yung mice at 3 months of age for both genotypes. Tissues were isolated from 30 month old mice for WT and 24 month old mice from Het animals. Western blots were probed with antibodies against the indicated proteins. Actin was probed as a loading control.

Supplemental Figure 3. Tissue-specific analysis of Hat1 and γ-H2AX during aging in $Hat1^{+/+}$ mice. A-D. The indicated tissues were isolated from young (3 month) and old (30 month) $Hat1^{+/+}$ mice. Tissue sections were stained with DAPI and with antibodies against Hat1 and γ-H2AX, as indicated. Images were taken at 10X magnification.

Supplemental Figure 4. Tissue-specific analysis of Hat1 and γ-H2AX during aging in $Hat1^{+/-}$ mice. A-D. The indicated tissues were isolated from young (3 month) and old (24 month) $Hat1^{+/-}$ mice. Tissue sections were stained with DAPI and with antibodies against Hat1 and γ-H2AX, as indicated. Images were taken at 10X magnification.

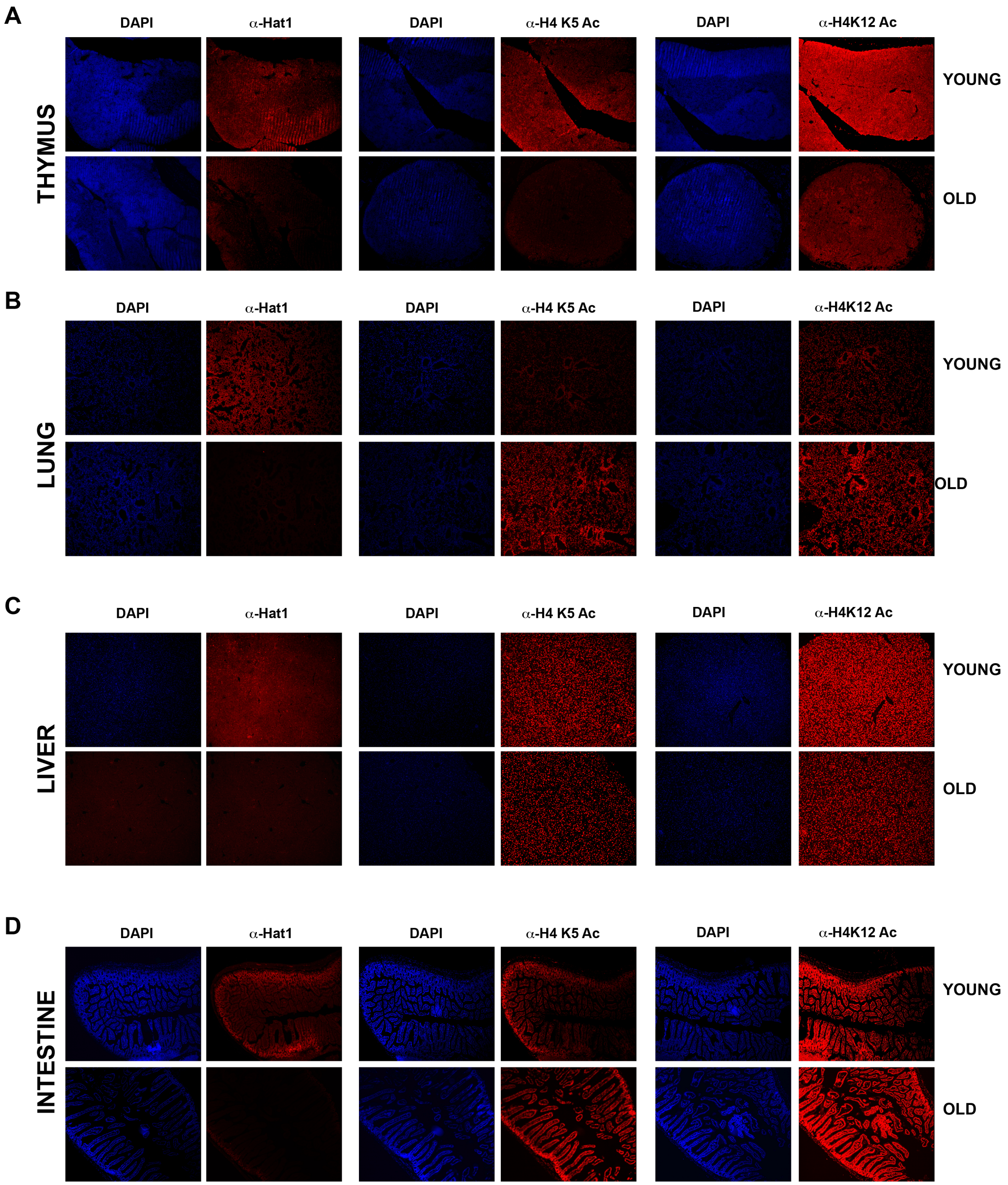

Supplemental Figure 1

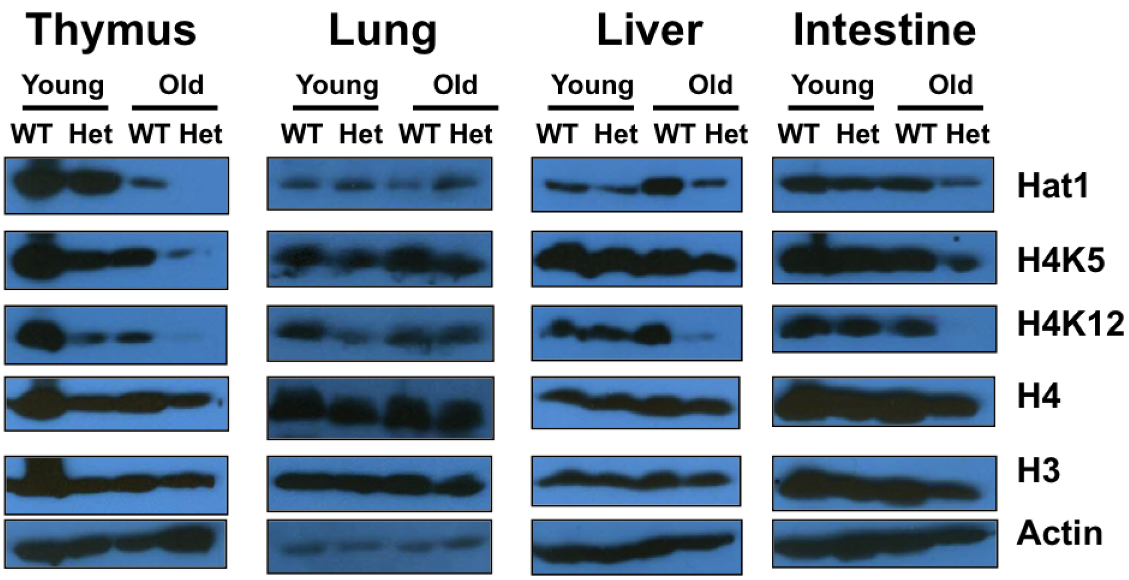

Supplemental Figure 2

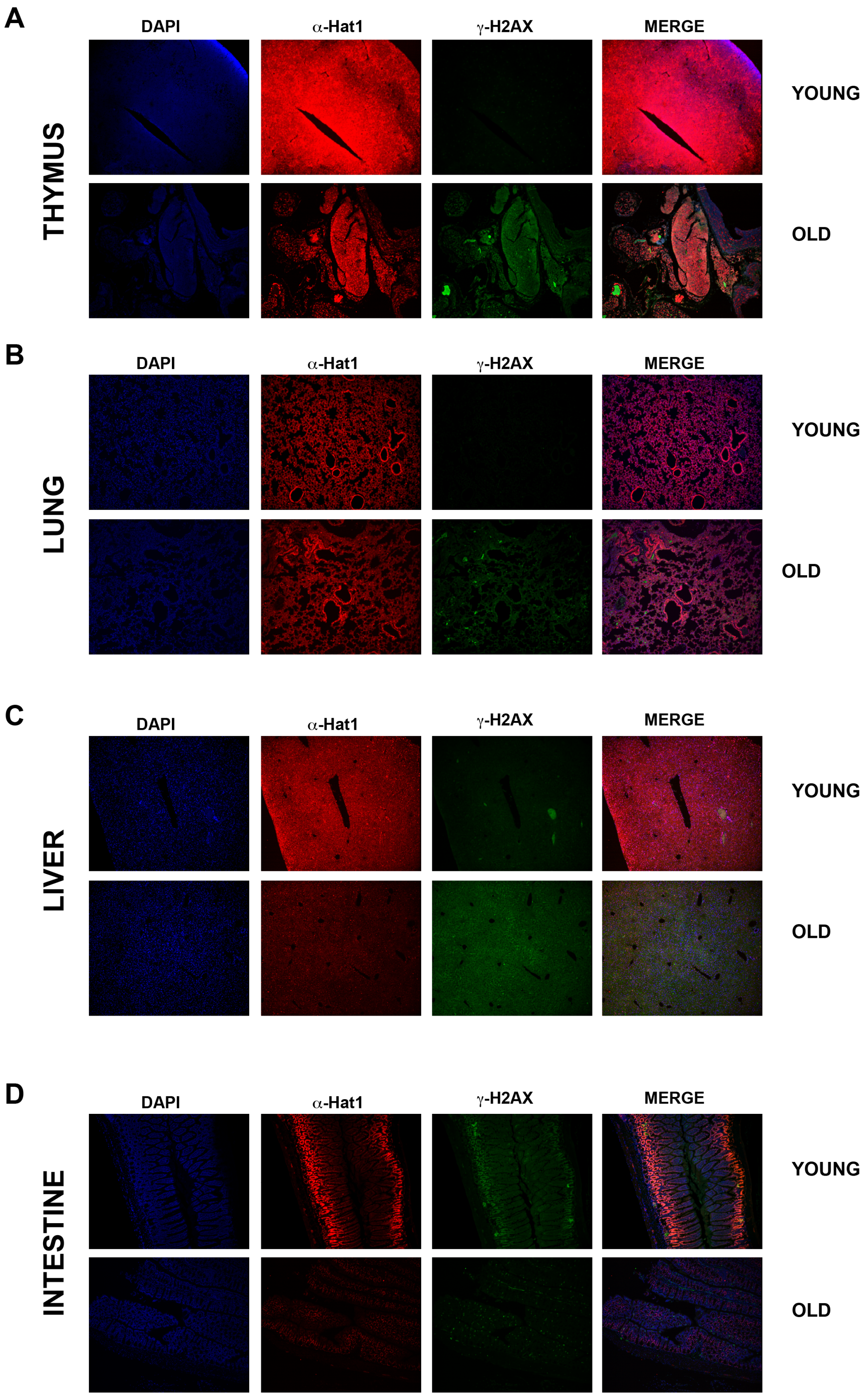

Supplemental Figure 3

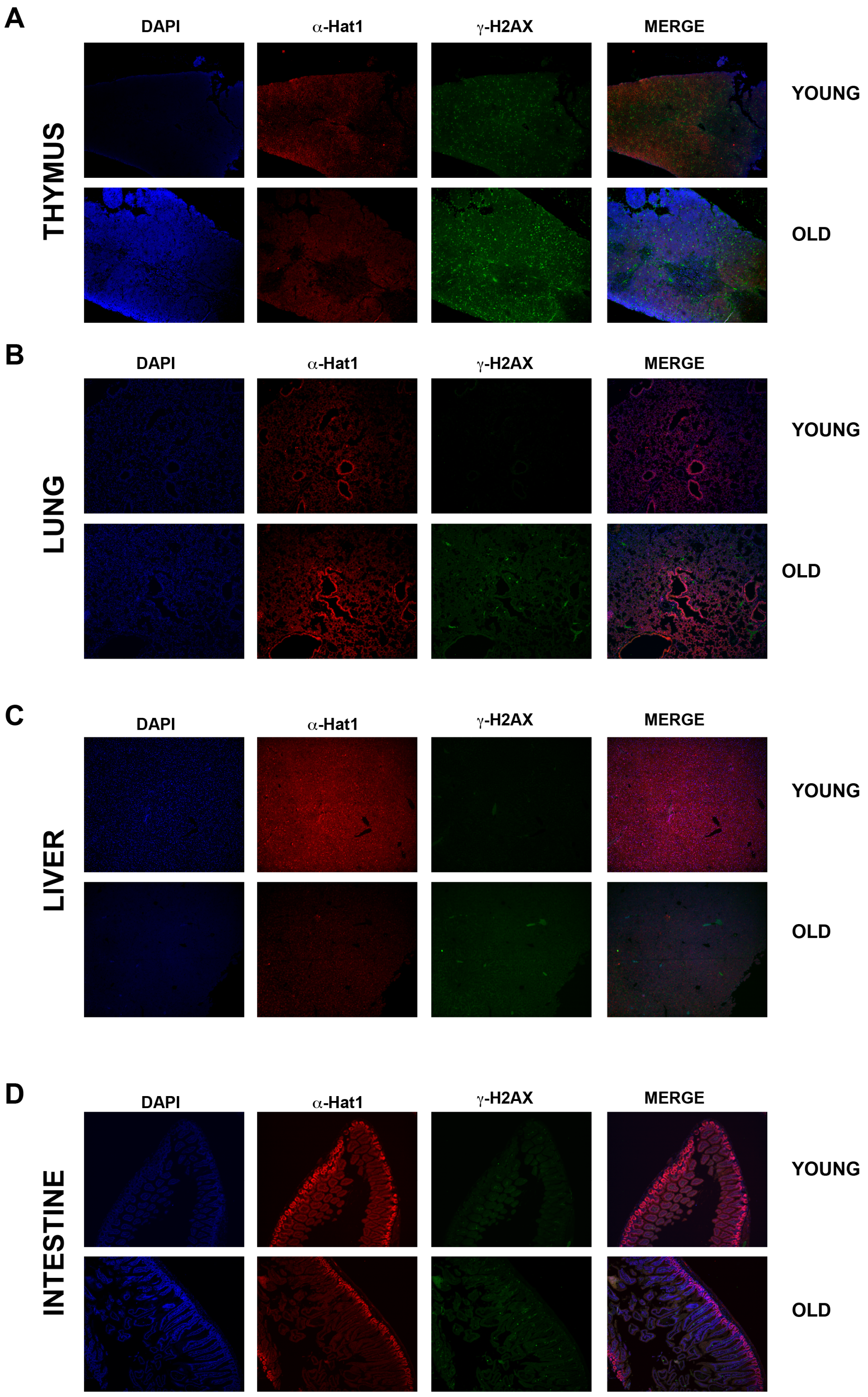

Supplemental Figure 4
